# Development and validation of a pipeline for the systematic search for new HLA alleles in WGS data

**DOI:** 10.3389/fbinf.2026.1751616

**Published:** 2026-04-07

**Authors:** Eugene Albert, Andrei Deviatkin, Daria Smirnova, Mary Woroncow, Gauhar Zobkova, Olga Mityaeva, Anna Smirnova, Viktor Bogdanov, Pavel Volchkov

**Affiliations:** 1 Lomonosov Moscow State University, Moscow, Russia; 2 Federal Research Center for Innovator and Emerging Biomedical and Pharmaceutical Technologies, Moscow, Russia; 3 Evogen LLC, Moscow, Russia; 4 Moscow Center for Advanced Studies, Moscow, Russia

**Keywords:** database, human leukocyte antigen alleles, IPD-IMGT/HLA, WDL pipeline, whole-genome sequencing

## Abstract

**Background:**

Human leukocyte antigen (HLA) is a highly polymorphic locus in the human genome that also has high clinical significance. New alleles of HLA genes are constantly being discovered but mostly by HLA typing laboratories using field-specific protocols, such as enrichment of the HLA region in high-throughput sequencing data. Nevertheless, a vast amount of whole-genome sequencing (WGS) data has been accumulated over the past years. The main goal of our work was to develop and validate a pipeline specifically tailored toward the identification and characterization of new HLA alleles from 30x WGS sequencing.

**Results:**

In this article, we present a pipeline, HLAchecker, which is specifically designed to identify potentially new HLA alleles based on discrepancies between predicted HLA types, generated using any other dedicated tool, and the underlying raw 30x WGS data. HLAchecker reports results in a structured way that simplifies further validation of potentially new HLA alleles and streamlines the submission of alleles to appropriate databases. We validated this tool on 4,195 30x WGS samples and 6 HLA genes (*A*, *B*, *C*, *DQA1*, *DQB1*, and *DRB1*) typed by HLA-HD and discovered 17 potentially new HLA alleles with substitutions in exonic regions. We further validated five of these alleles using Sanger sequencing and submitted them to the IPD-IMGT/HLA database.

**Conclusion:**

HLAchecker is suitable for the identification of new HLA alleles in large WGS cohorts accumulated by the scientific community in recent years. HLAchecker is freely available at https://gitlab.com/EugeneA/hlachecker.

## 1 Introduction

The human leukocyte antigen (HLA) genotype is an important feature of the human genome with various clinical implications, including susceptibility or resistance to certain diseases and donor–recipient compatibility for bone marrow transplantation. Therefore, HLA genotyping is widely used in routine clinical practice (Allen et al., 2018). HLA genotyping is performed by comparing the sample with a set of references. Experimentally, this can be performed using the polymerase chain reaction (PCR) (Nguyen et al., 2017) or high-throughput sequencing (Wang et al., 2012). In the clinical setting, the high-throughput sequencing approach is usually tailored to high-coverage sequencing of the HLA locus using specific HLA enrichment kits. The enrichment of HLA loci for sequencing with specific kits increases the accuracy of the analysis and reduces its price. Therefore, HLA typing in routine clinical practice today is performed either using HLA-locus PCR enrichment kits and subsequent sequencing or using a direct PCR test. It should be noted that both methods can be used to determine whether a patient has an allele that is similar to an allele contained in the reference database. If the patient has an HLA haplotype that does not match the allele in the reference set, the analysis would identify the HLA haplotype from the reference set that is most similar to the patient’s allele. Consequently, a new HLA haplotype which, by definition, is not included in the reference database, would not be correctly typed without special efforts and dedicated bioinformatics analysis. Nevertheless, the whole-genome sequencing (WGS) data collected over the years provide a good opportunity to systematically identify new HLA alleles and, therefore, improve overall HLA typing results. Moreover, WGS analysis provides an additional opportunity to fill the gaps in the currently deposited records, which, for historical reasons, often contain only partial (several exons) sequences of alleles.

Over the last 15 years, a number of tools have been developed to solve the task of HLA genotyping from WGS data (Lee and Kingsford, 2018; Kawaguchi et al., 2017; Kim et al., 2015). Such genotyping is a complicated process due to the high similarity between alleles of HLA genes and the sparse nature of the IPD-IMGT/HLA database, in which some alleles are only represented by partial CDS sequences. Some of the developed tools could be used in the in-house pipeline to recognize new HLA alleles from WGS data [e.g., Kourami, HISAT-genotype, or the more recent T1K (Song et al., 2023)]. However, none of these tools are directly tailored for the discovery and characterization of new alleles. For example, Kourami only reports the identity metric between the predicted allele and the assembled haplotype, which hints at the presence of a novel allele but leaves all post-processing analysis, including basic variant calling, to the user. HISAT-genotype produces a visual representation of the alignment between reference alleles in .pdf format and the assembled haplotype, along with a Binary Alignment Map (BAM) file of reads mapped to the hg38 reference. However, on one hand, a BAM file with reads mapped to the human reference sequence has very limited utility as it contains tens to hundreds of SNPs. On the other hand, the visual representation that potentially might be used for qualitative evaluation of the results is in .pdf format, not particularly suitable for either automatic or manual validation. However, more importantly, both of these tools have not been updated recently, and updating their references to the latest release of the IPD-IMGT/HLA database is a challenging task in itself but absolutely crucial for the correct typing of novel HLA alleles. T1K, as a more recent tool, has no issues with the database update and outputs a .vcf file containing variants in the typed allele. Nevertheless, T1K functionality neither covers post-calling characterization of the variant, which is required for the novel allele, nor provides aligned reads or other metrics for variant evaluation. Therefore, a lot of manual labor is still required to systematically screen large cohorts of samples for potentially new HLA alleles. As far as we know, the only tool specifically suited for this task—NovAT (Simakova et al., 2022)—is only available as a web application, which makes its implementation into a running analysis pipeline impossible and poses problems with sensitive data management. In the current study, we have developed a freely available tool to characterize HLA alleles based on 30x WGS data—HLAchecker (https://gitlab.com/EugeneA/hlachecker). HLAchecker operates on HLA typing results from any of the HLA typing tools and WGS-aligned BAM or Compressed Reference-oriented Alignment Map (CRAM) files. HLA typing results are evaluated against raw data, and a detailed report is generated for each case of discrepancy, which marks a potentially novel allele of the HLA gene. The report is specifically designed to expedite further validation and submission of the discovered allele to appropriate databases. We validated the tool on a cohort of 4,195 WGS samples typed with HLA-HD, along with 6 HLA genes (*A*, *B*, *C*, *DQA1*, *DQB1*, and *DRB1*), identified 17 potentially new HLA alleles, and validated 5 of these alleles using Sanger sequencing. Validated alleles were submitted to the IPD-IMGT/HLA database.

## 2 Materials and methods

### 2.1 Pipeline description and organization

All steps of data analysis are described in the *Results* section of the manuscript. Technically, the pipeline is written in Workflow Description Language (WDL), which brings all the advantages of specific pipeline-oriented frameworks, such as logical task-based structure, containerization of individual tasks with Docker, resource management, and batch submissions. HLAchecker takes aligned BAM or CRAM files and HLA typing results as inputs and outputs a report of potentially new HLA alleles. This two-step approach for HLA genotyping involves first using SAMtools (Li et al., 2009) (v1.18) to perform targeted read extraction from the 5 MB HLA region on chromosome 6, followed by sensitive remapping of these reads to polymorphic HLA gene references from the IPD-IMGT/HLA database (Barker et al., 2023) (v3.53 was used throughout the paper) using Minimap2 (v2.26) (Li, 2018).

A custom script written in Biopython is used to generate reference sequences and supplementary sequences (specified in the main text) for further use in the pipeline.

Bowtie2 (v2.5.3) (Langmead and Salzberg, 2012) is used to map selected reads to generated references. This tool was chosen because Bowtie2 was integrated into several widely accepted HLA typing tools [e.g., HLA-HD (Kawaguchi and Matsuda, 2020), OptiType (Szolek et al., 2014), RNA2HLA/seq2HLA (Chelysheva et al., 2021), and consHLA (Bowen-James et al., 2025)]. This extensive community adoption demonstrates its suitability for processing reads from the highly polymorphic HLA region. Concretely, for each read, it follows a four-step process: extracting seed substrings, performing ungapped alignment of these seeds using the full-text minute index, prioritizing seed hits, and finally extending the seeds into full alignments using dynamic programming. Bcftools (v1.19) (Danecek et al., 2021) is used to identify single-nucleotide variants and indels from the resulting BAM files for variant calling under a haploid assumption, executed with the parameters “call-mv-P 1.1e-2–ploidy 1” to set a mutation prior of 0.011 and output only variant sites.

A custom script written in Biopython is used to identify variants in exons and define amino acid substitutions. If variants in exonic regions are detected, BLASTN (Altschul et al., 1990) is used to search the obtained consensus sequences against a local copy of the NCBI Eukaryota nt database, which is subset for human sequences with blastdbcmd using the following command: “blastdbcmd-db nt_euk-taxids 9606-outfmt %f-out human.fa.” To allow BLAST alignment results to include official IMGT/HLA identifiers when a match is found (for example, if an outdated IMGT/HLA version is used for typing), a local copy of NCBI can be extended with sequences from IMGT/HLA using the blastdb_aliastool command. Mosdepth (v0.3.3) (Pedersen and Quinlan, 2018) is used to calculate mean coverage for each allele of each gene. IGV is used to visualize results for candidate cases. All tools are packed in Docker containers and uploaded to Docker Hub.

### 2.2 Cohort selection

To evaluate the possibility of discovering new HLA alleles based on standard 30x WGS data, we used samples collected by “Evogen” during routine sequencing of the healthy Russian population. We have selected WGS data for 4,195 individuals with a homogeneous genetic background based on principal component analysis (PCA), performed as described previously (Albert et al., 2023). A homogeneous genetic background was required for the correct analysis of HLA haplotype frequencies.

### 2.3 Library preparation and sequencing

Library preparation and sequencing were performed as described previously. In brief, DNA extraction was performed with a spin column using the QIAGEN QIAamp DNA Blood Mini Kit (Cat. No. 51106) from whole blood, according to the manufacturer’s protocol. DNA amount was measured fluorometrically using Qubit4 (Thermo Fisher Scientific)/DeNovix (DeNovix Inc.). For the subsequent library preparation, only high-quality genomic DNA (OD260/OD280 = 1.8–2.0, OD260/OD230 > 2.0) was used. Library preparation was performed using a PCR-free enzymatic fragmentation protocol (MGIEasy FS PCR-Free DNA Library Prep Set, Cat. No. 1000013455) with 800–1,200 ng of gDNA. The distribution of insert size was 400–600 bp. WGS library preparation was performed both manually and automatically. WGS was performed using DNBSEQ-G400 (MGI Tech Co., Ltd.) with FCL PE150 (Cat. No. 1000012555), FCL PE200 (Cat. No. 1000013858), and DNBSEQ-T7, according to the manufacturer’s protocol. Average sequencing depth was 30x.

### 2.4 Sanger validation

Five samples with detected potentially new HLA allele candidates were selected for validation via Sanger sequencing. Two pairs of allele-specific PCR primers were chosen for each sample using NCBI primer-blast software (Supplementary Table S1). Allele specificity was achieved by selecting primers specifically aligned to one allele and not to the other alleles predicted by HLA-HD, such that the SNPs predicted by HLAchecker fall close to the middle of the PCR product for accurate Sanger sequencing. Annealing temperatures were optimized for each primer pair (ranging from 60 °C to 65 °C, Supplementary Table S1). The cycle number was 30 for all reactions. The polymerase PrimeSTAR GXL DNA (TaKaRa Bio Inc., Japan) was used for the reaction. The Cleanup Standard Kit (Evrogene, Russia) was used for the gel purification of PCR products. Purified PCR products were sequenced using the BigDye Terminator Kit v3.1 and ABI 3500 Genetic Analyzer (Applied Biosystems, United States), according to the standard manufacturer’s protocols.

### 2.5 HLA typing and haplotype frequencies

HLA typing from WGS was performed using either HLA-HD (v1.7.0) (Kawaguchi and Matsuda, 2020) or T1K (Song et al., 2023), according to the authors’ recommendations. HLA haplotypes frequencies were calculated using Hapl-o-Mat (v1.2.2) (Schäfer et al., 2017), according to the authors’ recommendations.

### 2.6 Ethics approval

The study was approved by the local ethical committee of the Independent Multidisciplinary Committee on Ethical Review for Clinical Trials (Moscow, Russia) and was performed in accordance with the approved guidelines and the Declaration of Helsinki. Informed consent was obtained from all participants.

## 3 Results

### 3.1 Structure of the pipeline for the identification of new HLA alleles from WGS data

**FIGURE 1:**
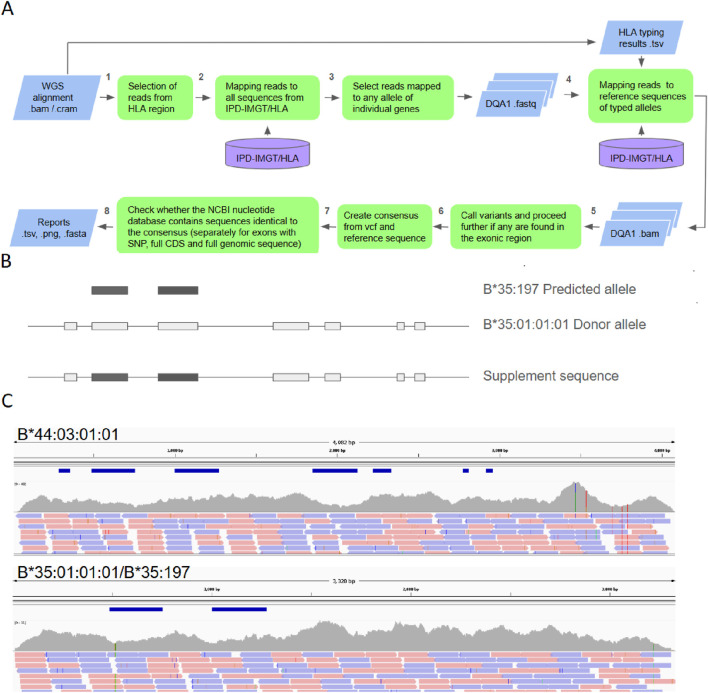
Schematic pipeline representation. **(A)** Overall pipeline schema. **(B)** Example of supplement sequence generation for alleles that lack a full gene sequence. Allele B*35:197 has only two exon sequences in IPD-IMGT/HLA. The absent genomic sequence is supplemented with the B*35:01:01:01 sequence (“Donor allele”). **(C)** Example reads mapped to gene B for a sample with defined haplotypes B*44:03:01:01/B*35:197, where the absent genomic sequence for B*35:197 is supplemented with B*35:01:01:01. Coverage, reads, and exonic regions track are shown. Note the two SNPs in the B*35:197 exon.

The overall structure of the pipeline for identifying potential new alleles of HLA genes with variations in exonic sequences is shown in Figure 1A. This pipeline takes the results of whole human genome sequencing alignment in BAM or CRAM format and HLA typing results from any dedicated tools as input and produces a report of potentially new HLA alleles as output. Only reads mapped to the 5 Mb locus of the sixth chromosome and to the alternative contigs of the sixth chromosome, which contain the HLA locus, are used for further processing (step 1 in Figure 1A). Filtered reads were mapped to all possible reference genomic sequences from the IPD-IMGT/HLA database (step 2 in Figure 1A). Only reads mapped to the selected HLA genes (*DQA1*, *DQB1*, *DRB1*, *A*, *B*, and *C*) were used for further analysis (step 3 in Figure 1A). These reads were used for mapping to the genomic sequence of the alleles for each given gene predicted using the external HLA typing tool (step 4 in Figure 1A). In this study, HLA-HD was used for typing HLA for both alleles in each sample.

It should be noted that, for some HLA alleles, the IPD-IMGT/HLA database lacks the full genomic sequence. In these cases, HLAchecker creates an artificial supplementary sequence with the missing part of the HLA allele. The artificial sequence is created by supplementing the missing part of the allele of interest with genomic sequences of the closest allele based on the HLA nomenclature (Figure 1B). The selection of the closest allele is performed by sequentially lowering the allele resolution until the first entry with a full genomic sequence is found in the IPD-IMGT/HLA database.

Mapping (step 4 in Figure 1A) was performed separately for each gene and a set of selected reads. Both alleles of a selected gene were used simultaneously as a reference. This approach allows automatic phasing of alleles (Figure 1C). For individuals harboring HLA pseudogenes in their genome, direct mapping without any post-processing of the results produces noisy alignments. Supplementary Figure S2 demonstrates mapping results of NovAT, HLAchecker without any mapping post-processing, and HLAchecker with filtering reads harboring more than two mismatches. It is evident that filtration greatly reduces noise in the resulting data for complicated cases and evens the coverage over the gene length. We characterize the distribution of variant quality metrics (depth and Phred) discovered with different thresholds on the number of mismatches in individual reads to select optimal filtration parameters (Supplementary Figure S3). Notably, allowing more mismatches increases the number of artifact variants—with extremely high coverage or low Phred. For the analysis in this study, we selected a more conservative threshold of 2 to reduce the number of false-positive discoveries. To allow users of HLAchecker more flexibility, we also made it possible to adjust this threshold through the pipeline inputs. The generated BAM files were used for SNP/INDEL calling (step 5 in Figure 1A). If any variants are called in the exonic region of the HLA allele, then further analysis is conducted. First, genomic consensus sequences were generated for the allele with the discovered variant. At that stage, both exonic and intronic variants were used (step 6 in Figure 1A). It should be noted that the IPD-IMGT/HLA database is a strictly curated database and, therefore, updates slowly compared to other databases for sequence deposition, e.g., GenBank. Therefore, it is possible to falsely identify sequences that are not included in the latest version of IPD-IMGT/HLA but are already described in another database as a new allele. Therefore, an additional comparison of the obtained consensus with the less stringent NCBI Nucleotide Database was performed (step 7 in Figure 1A). The NCBI Nucleotide Database contains partial allele submissions, along with full alleles. Therefore, three BLASTN comparisons were performed for each candidate consensus separately: for the exon with the SNP, the full CDS consensus sequence, and the full genome consensus sequence. At the final step, HLAchecker generates reports with information specifically tailored for the submission of discovered alleles to GenBank and IMGT. In addition, screenshots of the mapping of reads to references are generated and visualized using the Integrative Genomics Viewer for each allele with exonic variation to allow easy manual review (step 8 in Figures 1A,C).

### 3.2 Structure of HLAchecker output tailored for the submission guidelines of IPD-IMGT/HLA

If an SNP was identified in the exonic region of a predicted allele, HLAchecker produces the report and several associated files for the evaluation of potential findings. The report contains several sections that are designed to simplify allele characterization and submission to the appropriate database. The report contains the following sections:

- Alleles—a list of the two haplotypes for the specific HLA gene analyzed in the current report.
- Variants—a list of exonic/all variants from the .vcf file, identified in the given sample.
- Protein alignment—link to the allele in the IPD-IMGT/HLA database, amino acid change, protein position at which changes occur, affected exon, and alignment of the protein sequence of the originally typed allele to the sequence with identified variants.
- Blast results for the exon with SNP—top five results of three BLASTN comparisons of the consensus sequence (exon with SNP, CDS, and genomic) to the NCBI database (local copy).
- HLA typing results—the complete HLA typing input for the sample, which includes results for all HLA genes. These data are either supplied by the user or generated internally by HLA-HD if no external typing is provided.
- Exon consensus sequence, CDS consensus sequence, and full gene consensus sequence—consensus sequences of corresponding entities.

Apart from the textual report, HLAchecker produces .bam files of reads mapped to the sequence of the typed HLA gene, .png screenshots from IGV for quick visual control of mapping, and the associated .fasta gene reference.

### 3.3 Developed pipeline successfully identifies discordance between the sequences of predicted HLA alleles and WGS data

The purpose of HLAchecker is to identify and automatically characterize discordance between raw WGS data and the prediction of the HLA typing tool for a given sample. To simulate such discordance and estimate pipeline efficiency, we introduced random exonic SNPs in reference sequences of the HLA allele prior to mapping selected reads using Bowtie2 (Figure 1A, step 4). The introduction of changes into the reference database is an appealing strategy for testing the capability of a tool to identify novel alleles as it bypasses problems associated with *in silico* generation of raw reads for novel alleles. Such a strategy was successfully applied, for example, for similar purposes during the Kourami description. We selected 100 random samples from the cohort where the initial run of the pipeline did not identify any HLA-associated mismatches. We repeated the analysis and introduced one random exonic SNP in the reference sequence of each HLA allele for each sample for six main HLA genes: *A*, *B*, *C*, *DQA1*, *DQB1*, and *DRB1*. The pipeline successfully identified 1,105 out of 1,135 (not 1,200 because some samples have homozygous alleles) artificially introduced SNPs, demonstrating that 30x WGS data on average harbor enough reads to correctly identify samples with new HLA alleles.

We manually examined the SNPs that were missed by HLAchecker. They were all located in regions that are identical between two alleles, resulting in mapping of the reads to the paired unmodified reference sequence (Supplementary Figures S4A,B).

We repeated the introduction of random SNPs in the same set of samples four more times. The averaged results presented in Table 1 show that the average sensitivity of our approach is 97%, ranging from 95.3% (C) to 98.8% (DQA1) across individual genes. It should be noted that such a simulation design does not allow the calculation of the number of false positives and true negatives.

**TABLE 1:** Contingency table demonstrating HLAchecker sensitivity. FP averaged across five independent simulations.

Status	Predicted positive	Predicted negative
Actual positive	True positive = 1,135	Mean false negative = 34
Actual negative	False positive = N/A	True negative = N/A

### 3.4 HLA allele frequencies in the cohort used for HLAchecker validation were consistent with publicly available population data

**FIGURE 2:**
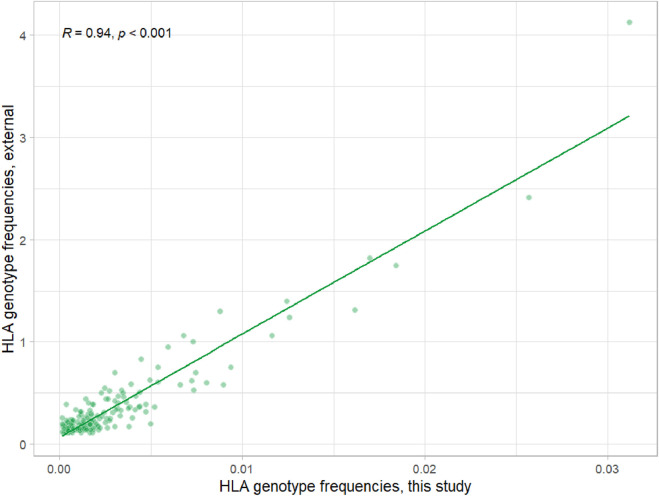
Correlation of frequencies for A-B-DRB1 haplotypes between data from www.allelefrequencies.net for the central Russian population (Pukhlikova et al., 2015) (measured in percent) and frequencies from current studies.

To validate HLAchecker on the real 30x WGS data, we selected a cohort of people from a dataset of LCC Evogene, which was obtained through routine 30x WGS of healthy individuals. A population analysis and filtering were performed to prevent a possible enrichment of the cohort with the new alleles and the associated bias. The cohort was restricted to individuals of Central Russian ancestry, which is well characterized in terms of the frequency of HLA alleles. In total, the genomic information of 4,195 individuals was used (Supplementary Figures S5). HLA typing of the cohort was performed using the HLA-HD tool, and subsequent analysis was restricted to the major HLA genes: *A*, *B*, *C*, *DQA1*, *DQB1*, and *DRB1*. To validate the obtained HLA-HD results using an orthogonal method, we compared the frequencies of haplotypes in the selected cohort with data from the www.allelefrequencies.net database for the Moscow population (Figure 2; Supplementary Table S2). Haplotypes and their frequencies were inferred from HLA-HD results using Hapl-o-Mat software, according to guidelines. Overall, the results of HLA typing with HLA-HD showed a good correlation with the independent dataset created using a different method. Therefore, the results obtained from our cohort can be used for the further validation of HLAchecker.

It should be noted that this population-level frequency comparison was performed to validate the representativeness of our cohort, not to reassess the accuracy of the HLA-HD typing tool. The concordance with published population data confirms that our cohort is suitable for novel allele discovery and that any identified variants are likely to reflect real genetic diversity rather than cohort-specific artifacts.

### 3.5 Analysis of the 4,195 30x WGS samples revealed 17 potentially new HLA alleles

To evaluate the capability of HLAchecker to identify new alleles in 30x WGS data, we ran it on the aforementioned cohort. A statistical summary of the results is provided in Table 2. We categorized cases where HLAchecker detected a potentially novel allele into three categories according to information in the NCBI nt database: full genomic sequence, CDS sequence, or sequence of an exon with the variant absent from the database. As the data show, full genomic sequences are often missing in the NCBI db, but this is not a robust indicator of a novel allele as its CDS or an individual exon with the variation could already be reported. This observation highlights two facts. First, many alleles not yet present in the strictly curated IPD-IMGT/HLA database have already been deposited in other, less restrictive public repositories. Second, many alleles deposited in IPD-IMGT/HLA lack accompanying full-length genomic or CDS sequences. Detailed statistics for counts of incomplete HLA alleles in the cohort are present in Supplementary Table S3. Overall, 731 persons out of 4195 carry at least one HLA allele, which has no genomic sequence in the IPD-IMGT/HLA database. We estimated novel allele frequencies in the cohort based on the most stringent cases, in which the exon sequence with the variant is absent from the NCBI db (17 cases in total). The frequency of previously unknown exon sequences [n = number_of_new_sequences/(2*cohort_size)] varied for the six major HLA genes, ranging from 0.00024 for B and C to 0.00071 for DQA1.

**TABLE 2:** Summary of analysis of 4,195 samples for discrepancies between sequences of predicted HLA alleles and WGS data. Selected samples with exonic variant coverage above 5.

Gene	The number of unique alleles with SNP(s) in exon(s) (potentially new alleles)	The number of unique potentially new alleles in which the exon with the variant is absent from the NCBI nucleotide database	The number of unique potentially new alleles in which the protein coding sequence is absent from the NCBI nucleotide database	The number of unique potential new alleles in which the full genomic sequence is absent from the NCBI nucleotide database
A	103	2	7	17
B	81	2	17	33
C	90	3	20	32
DQB1	6	2	2	3
DQA1	17	6	11	12
DRB1	22	2	11	19
TOTAL	319 (in 380 samples)	17	68	116

Sanger sequencing is the gold standard for the validation of any alteration in DNA; therefore, a set of variants identified in the cohort by HLAchecker was validated using Sanger sequencing. We randomly selected five samples with the exonic sequences absent from the NCBI Nucleotide Database for validation (Table 3). For each sample, two pairs of allele-specific primers were selected, which selectively amplify either the exonic region from the allele with the variant or the corresponding region from the non-altered allele (Supplementary Figure S1; Supplementary Table S1). Sanger sequencing of PCR products was performed in both directions from the same primers according to the submission guidelines of the IPD-IMGT/HLA database. All five new allelic variants were validated through Sanger sequencing and submitted to the IPD-IMGT/HLA database. Sanger sequencing results are summarized in Table 3. Raw Sanger sequencing data are presented in the Supplementary Materials. HLA haplotypes for patients with new HLA alleles are presented in Supplementary Figure S4.

**TABLE 3:** Summary of pipeline prediction validations with Sanger sequencing.

Sample	Allele	SNP position in CDS	Substitution in the nucleotide sequence	Substitution in the protein sequence	Exon with the variant
4,122	HLA00613 DQA1*05:01:01:01	169	C–A	Q–K	2
5,362	HLA00434 C*07:02:01:01	828	A–C	Synonymous	4
2,363	HLA00723 DRB1*08:01:01:01	567	G–A	M–I	3
3,150	HLA00756 DRB1*11:04:01:01	429	C–T	Synonymous	3
2,743	HLA00601 DQA1*01:01:01:01	672	G–A	Synonymous	3

### 3.6 HLAchecker output is robust across different HLA typing results

To validate our approach against other tools that could mark the presence of new alleles, we chose T1K as the most recent allele. As a first step, we reanalyzed 380 samples where potentially new alleles were previously identified (Table 2) with T1K using the same version of IPD-IMGT/HLA as for the initial analysis with HLA-HD and HLAchecker. Because T1K outputs only vcf files without any further processing, here we treated all alleles with identified SNPs as potentially new, without additional validation against sequence databases. Surprisingly, only 47 samples were identified as harboring potentially new alleles with 71 SNPs in total. From these SNPs, 51 (72%) were also identified using HLAchecker. We hypothesize that the huge difference in new allele estimation could be attributed to initial differences in HLA results. Therefore, we reanalyzed same 380 samples with HLAchecker, using HLA typing results from T1K as input instead of HLA-HD results. A change in the HLA typing results reduces the number of samples with the identified potential new allele from 380 to 84, demonstrating the dramatic impact on the overall results. Over the selected 380 samples and 6 genes, 563 alleles were typed differently between HLA-HD and T1K using the same IPD-IMGT/HLA version. In other words, 12% of all alleles were classified differently using HLA-HD and T1K.

First, we hypothesized that the difference between the two tools to a large extent may be explained by the fact that we compared them on a challenging subcohort where HLAchecker identified imperfect agreement between the HLA-HD prediction and the raw data. It was also previously reported that T1K and HLA-HD HLA predictions align well with each other (Schaschl et al., 2025). Therefore, a second comparison of these tools was performed on a subcohort of 100 samples that we previously used for pipeline validation. There were only 31 allelic discrepancies between tools for 100 samples and 6 genes, which is substantially lower than in the previous comparison—12% versus 2.6% of all alleles.

Second, we reasoned that even though there is a substantial difference in the number of identified SNPs between runs of HLAchecker with different HLA typing inputs, overall results might still converge after checking against the sequence database (step 7, Figure 1A). The number of identified novel exonic sequences was 16 for the T1K input, and all these sequences were also among the 17 (Table 2) identified using the HLA-HD typing input. The only sample that harbors novel alleles based on one analysis and not on the other is shown in Supplementary Figure S6. Both typing tools identified one allele of the DQA1 gene as 01:02:02:01, but the second allele was 01:01:01:07 in the case of T1K and 01:01:01:01 in the case of HLA-HD. As shown in the figure, both haplotypes lead to imperfect read mapping, but only the HLA-HD output results in exonic SNPs and, therefore, triggers HLAchecker to report a discrepancy in the data.

These results demonstrate that although the choice of the HLA typing tool can influence intermediate predictions, the final output of HLAchecker remains remarkably consistent. The near-complete overlap (16 out of 17) of novel exonic sequences identified using two different typing tools as input suggests that HLAchecker is robust to variations in HLA typing outputs.

## 4 Discussion

In this study, we demonstrated the possibility of identifying new HLA alleles from standard 30x WGS human sequencing samples and presented a pipeline specifically tailored for that purpose. Such an approach can help expand the HLA allele repertoire by reanalysis of the HLA region in large cohorts of existing whole-genome sequencing data [e.g., GnomAD (Karczewski et al., 2020) and UK Biobank (Sudlow et al., 2015)]. Such an approach can also help fill the gaps in the IPD-IMGT/HLA database, supplementing missing full genomic sequences, which pose a serious problem in the HLA analysis. It should be noted that although reanalysis of accumulated short-read 30x WGS could provide a good way of high-throughput screening for new alleles, it does not have 100% accuracy in all cases, and independent validation (e.g., Sanger sequencing) is required for identified candidates.

We reanalyzed HLA typing results produced by HLA-HD in a cohort of 4,195 individuals from central Russia to estimate the frequency of new alleles in this relatively well-characterized population. In total, we identified 319 alleles of 6 genes that had mismatches with predicted sequences from IPD-IMGT/HLA and the coverage of identified SNP above 5. We also performed additional searches of the identified sequences in the NCBI Nucleotide database. From a total of 319 alleles with variants, 116 complete gene sequences, 68 complete protein coding sequences, and 17 exons with variants were absent from the NCBI Nucleotide database. Therefore, even in a relatively well-characterized population that is genetically similar to Europeans, reanalysis of a cohort comprising 4,195 individuals yields a substantial amount of valuable information. Five of the 17 HLA alleles with new exonic variants that were absent from both IPD-IMGT/HLA and NCBI Nucleotide databases were selected and validated using Sanger sequencing.

One of the main problems with HLA analysis tools is the need to constantly update the database. Minor changes in the database format cause scripts that generate input for the tools to become obsolete. For example, launching a popular tool for HLA typing, Kourami (Lee and Kingsford, 2018), with the newest IPD-IMGT/HLA release requires manual correction of the files. The pipeline presented here takes raw FASTA files of complete protein-coding and gene sequences from the IPD-IMGT/HLA database, providing most convenient way to keep data up to date for the analysis. Moreover, all steps of the pipeline are containerized with Docker, making it robust and easy to launch.

Several limitations, nevertheless, are associated with the proposed approach. First, it relies on HLA typing performed using some external tools. False-positive calls may arise from inaccurate typing or the version mismatch between the IPD-IMGT/HLA database used for typing and that used in the pipeline. Some individuals harbor pseudogenes in their genome, and reads from these regions might interfere with the HLAchecker processing, as well as with upstream HLA typing. Due to the sparse nature of the IPD-IMGT/HLA database and the presence of substantial amounts of alleles without full genomic sequences, we choose not to report variation in the intronic regions, which limits the application of our approach to the third field of HLA nomenclature. As was shown, a large difference in the results of new allele identification analysis could arise from the use of different HLA typing methods (T1K and HLA-HD in this article).

Simulation-based evaluation demonstrates high sensitivity for exonic SNP detection (97%), and the successful validation of all five selected candidates by Sanger sequencing indicates high specificity. In addition to analytical precision, the study design plays a significant role. Although analysis of 4,195 samples enabled the identification of several new alleles, the size of this cohort limits the detection of variants with a low population frequency. Furthermore, as the samples were selected from a single population, they do not reflect the full spectrum of HLA diversity in humanity. Therefore, to comprehensively characterize the rare allelic repertoire and obtain representative frequency estimates, studies involving significantly larger and more ethnically diverse cohorts, such as the UK Biobank, are required.

The HLA typing method is especially important for the task of new allele identification and characterization because the new alleles, which are absent from the IPD-IMGT/HLA database, specifically challenge typing tools. We showed that HLA-HD and T1K had different predictions in 2.6% of all alleles tested in the cohort of 100 samples, whereas HLAchecker found no discrepancies between HLA-HD results and the raw data. Nevertheless, in the cohort of 380 samples, where HLAchecker identified potentially new alleles based on HLA-HD data, HLA typing results of HLA-HD and T1K differ in 12% of all alleles tested. Furthermore, despite substantial differences in HLA typing inputs, the novel exonic sequences predicted using HLAchecker were almost identical. These results underline an important advantage of HLAchecker—its ability to work with outputs of any HLA typing tools providing users with the freedom to choose the best possible option. The cohort of HLA typing tools is constantly expanding, and newer options arise, including even the combination of several approaches in a single framework (Lai et al., 2024).

Overall, HLAchecker, as presented in this publication, provides a straightforward approach for the use of accumulated WGS datasets for the systematic expansion of the known HLA allele repertoire. HLAchecker is specifically tailored to providing all necessary information on the detected event, e.g., new the allele consensus sequence, positions of variants in the genomic sequence, amino acid changes, the number of exons affected by the variants, and results of BLAST analysis of the consensus sequence against NCBI Nucleotide databases. In total, this information substantially simplifies the subsequent steps of allele characterization—Sanger sequencing validation, submission to GenBank, and submission to IPD-IMGT/HLA.

## Data Availability

Allele frequencies available in the Supplementary table 2. Validated HLA alleles sequences available in GenBank (PP049249, PP763745, PP763746, PP763747, PP763748).
