# Supplementary material for: Development and validation of a pipeline for the systematic search for new HLA alleles in WGS data
Source: Front Bioinform. 2026 Apr 7;6:1751616. doi: 10.3389/fbinf.2026.1751616 (PMC13095684; doi:10.3389/fbinf.2026.1751616)
Supplement: Supplementary file 1 [file DataSheet1.zip › supplementaryMaterials/frontiers_supplementary materials_2.pdf]

Supplementary Material

1 SUPPLEMENTARY DATA

2 SUPPLEMENTARY TABLES AND FIGURES

2.1 Supplementary tables captions

Supplementary Table S1. Primer sequences for 5 validated new HLA alleles. (xlsx)

Supplementary Table S2. HLA alleles frequencies over investigated cohort. (tsv)

Supplementary Table S3. Summary statistics of HLA alleles without full genomic sequence in the IPD-IMGT/HLA database. (xlsx)

Supplementary Table S4. Full HLA haplotypes of 5 samples with validated new HLA alleles. (xlsx)

2.2 Supplementary figures

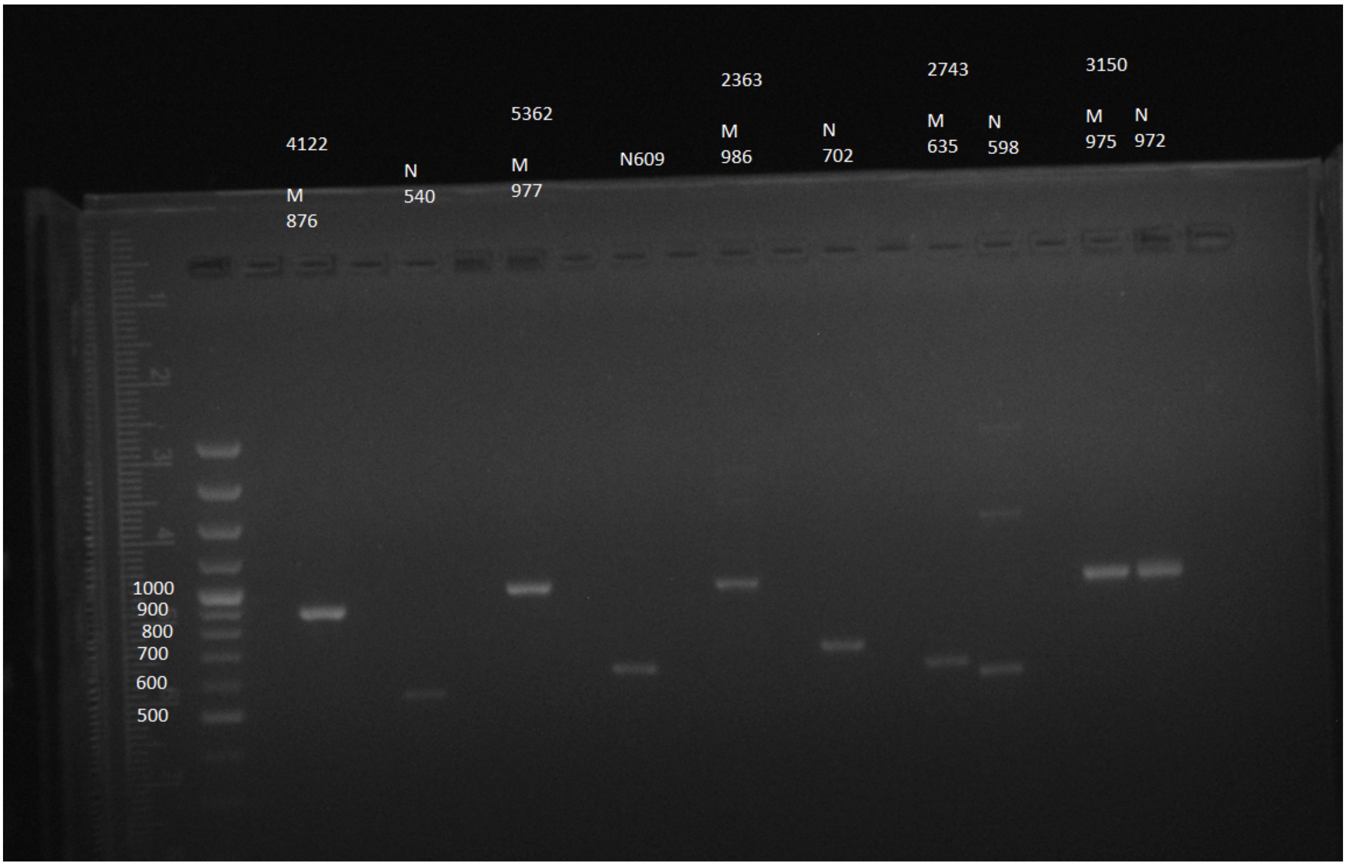

Figure S1. Allele specific PCR of five samples for fragments generation and subsequent Sanger sequencing.

A

B

C

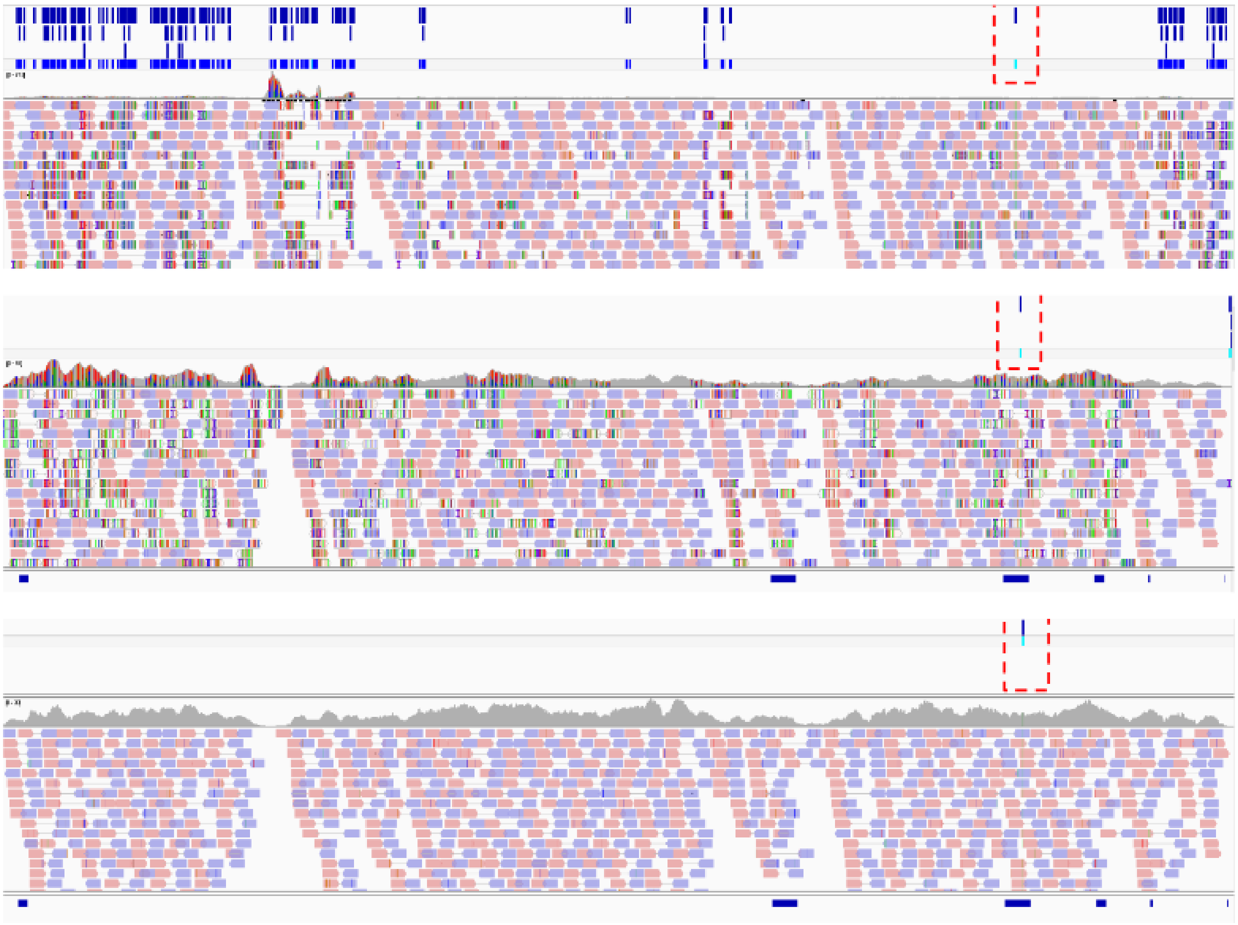

Figure S2. Reads filtration based on num- ber of mismatches reduce overall noise in the resulting align- ment and variant calling. IGV images represent same raw data for DRB1*08:01:01 allele. Underline variant was validated by Sanger sequencing. A. Resulting alignment and calling from NovAT tool (https://novat.parseq.pro/). B. HLAchecker without any filtra- tion by number of mismatches in reads. C. HLAchecker with mis- matches threshold set to 2 that was used throughout the paper.

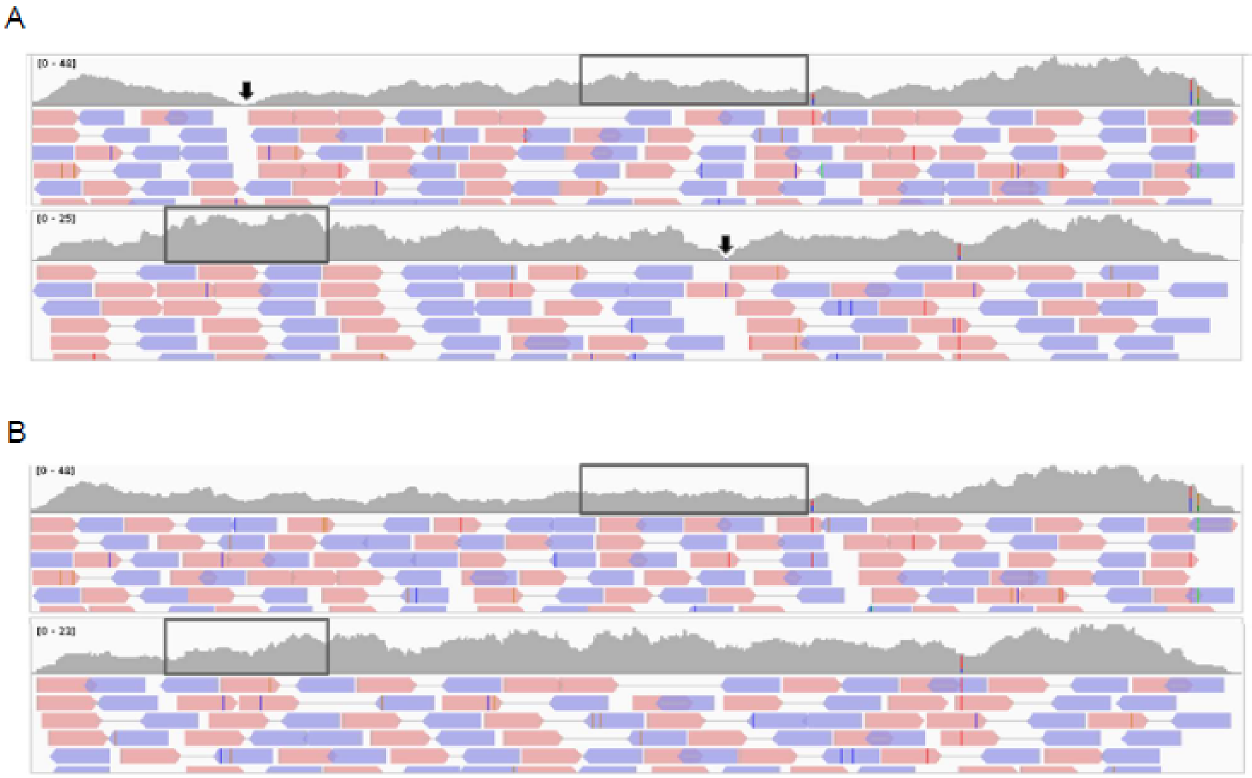

Figure S3. Distribution of variant quality met- rics (depth and Phred quality score) for different thresholds on number of allowed mismatches in the reads. Results for randomly selected set of 100 samples. A. Depth distribution. B. Phred distri- bution.

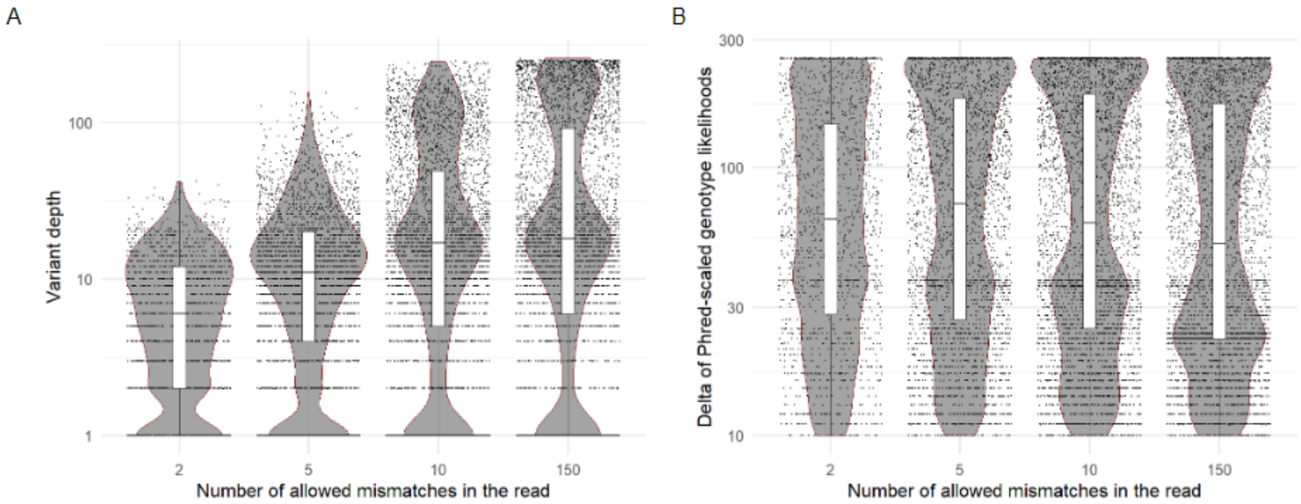

Figure S4. Example of false negative call for HLAchecker during in-silico validation. A. Mapping to both alleles of C gene is present. Random SNPs positions are marked with an arrow. B. Same sample as on A but no random SNPs were introduced. Note two gaps in the coverage around SNPs and changes in boxed coverage of corresponding alleles between A and B.

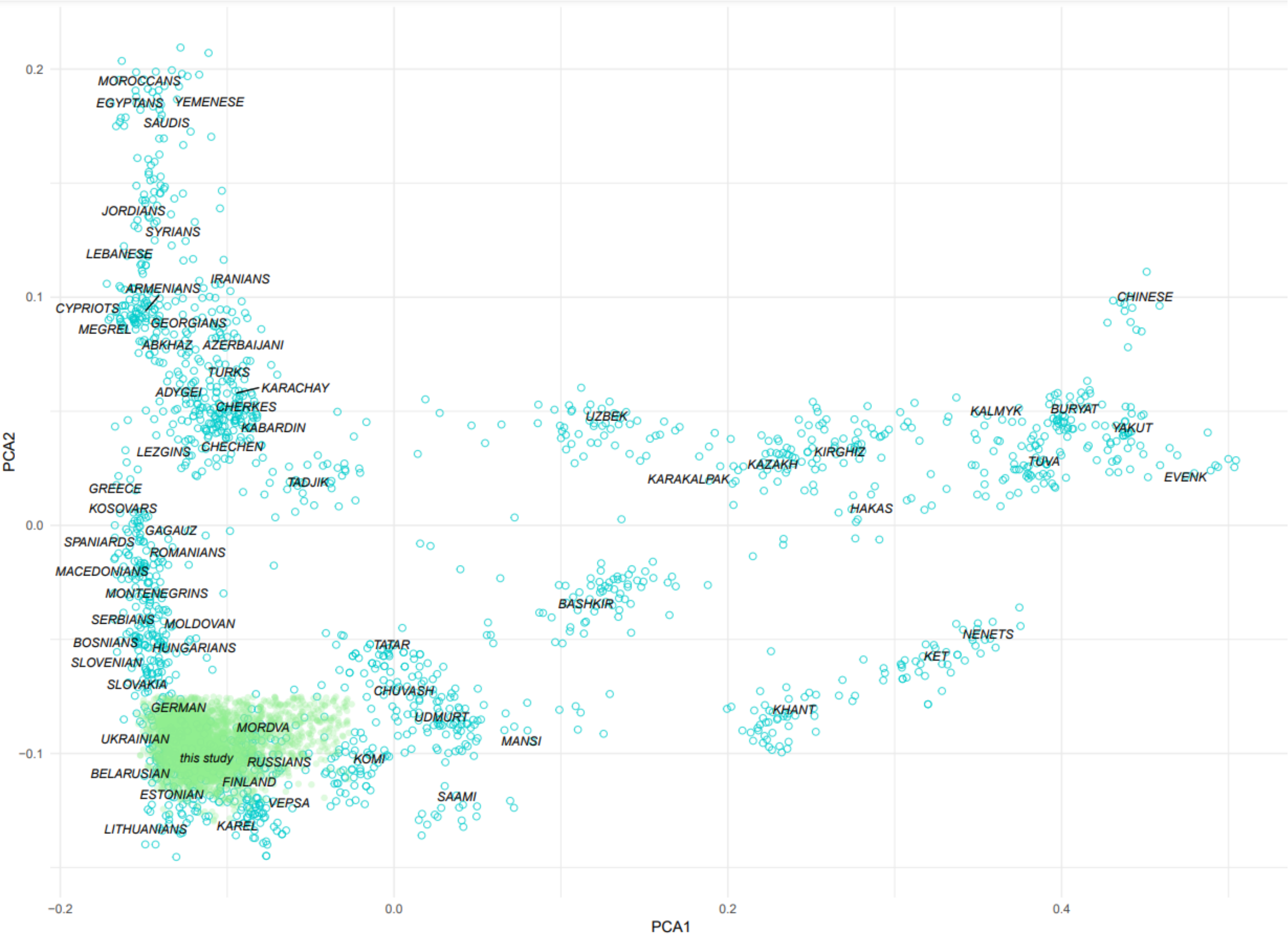

Figure S5. Ethnic analysis of the cohort used in the study. Blue circles represent the reference population, green circles represent cohort from the current study. PCA is built on the 40000 SNPs.

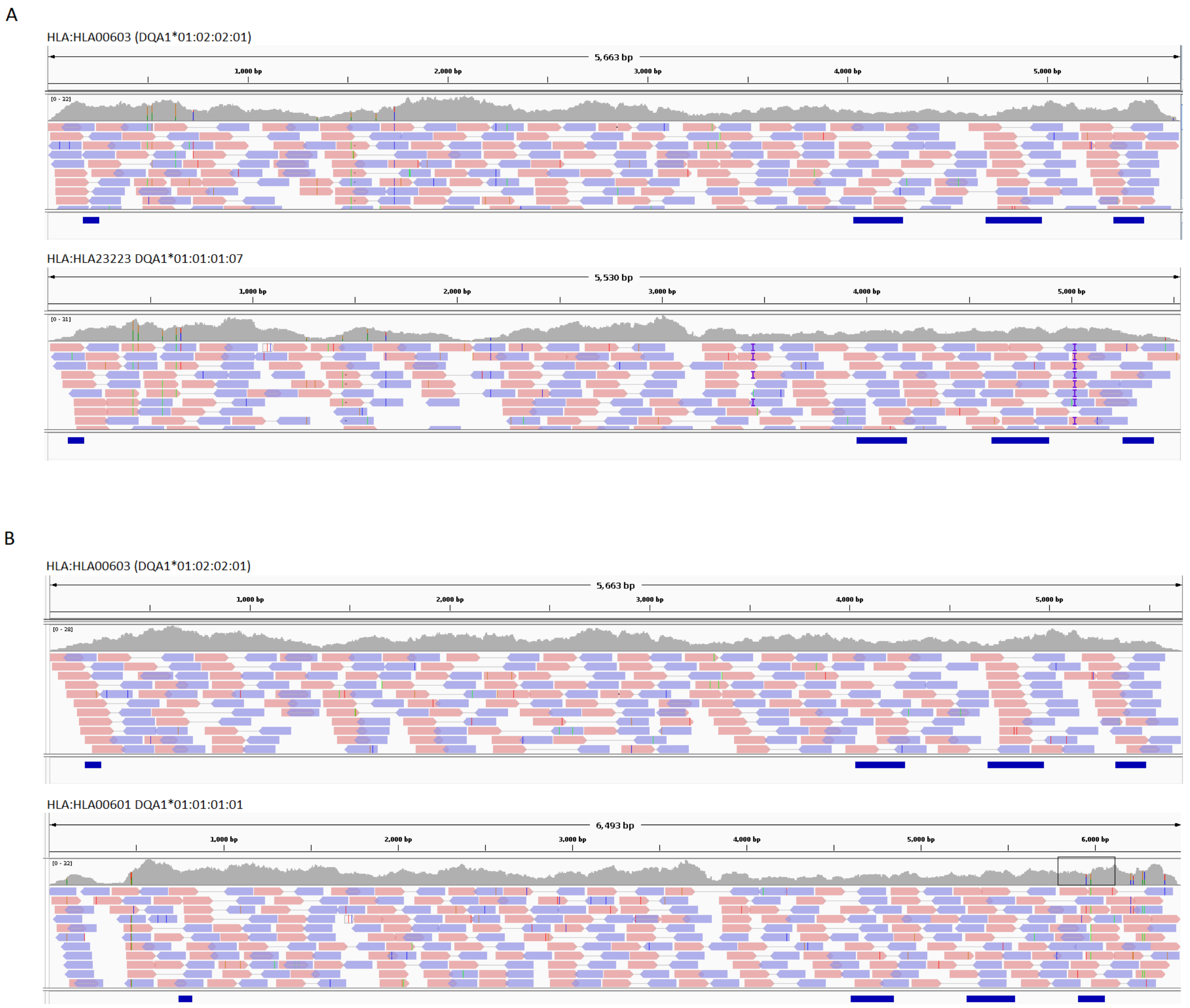

Figure S6. Reads alignment for sample where HLAchecker identified novel exonic sequence based on HLA-HD results and not on T1K results. A. Alignment on alleles predicted by T1K. B. Alignment on alleles predicted by HLA-HD, note exonic variants marked by black box.
